# RNA degradation in human mitochondria: the journey is not finished

**DOI:** 10.1093/hmg/ddae043

**Published:** 2024-05-23

**Authors:** Giulia Santonoceto, Aneta Jurkiewicz, Roman J Szczesny

**Affiliations:** Institute of Biochemistry and Biophysics, Polish Academy of Sciences, Pawińskiego 5A, Warsaw 02-106, Poland; Institute of Biochemistry and Biophysics, Polish Academy of Sciences, Pawińskiego 5A, Warsaw 02-106, Poland; Institute of Biochemistry and Biophysics, Polish Academy of Sciences, Pawińskiego 5A, Warsaw 02-106, Poland

**Keywords:** mitochondrial RNA decay, polyadenylation, mitochondrial gene expression, mitochondrial RNA surveillance

## Abstract

Mitochondria are vital organelles present in almost all eukaryotic cells. Although most of the mitochondrial proteins are nuclear-encoded, mitochondria contain their own genome, whose proper expression is necessary for mitochondrial function. Transcription of the human mitochondrial genome results in the synthesis of long polycistronic transcripts that are subsequently processed by endonucleases to release individual RNA molecules, including precursors of sense protein-encoding mRNA (mt-mRNA) and a vast amount of antisense noncoding RNAs. Because of mitochondrial DNA (mtDNA) organization, the regulation of individual gene expression at the transcriptional level is limited. Although transcription of most protein-coding mitochondrial genes occurs with the same frequency, steady-state levels of mature transcripts are different. Therefore, post-transcriptional processes are important for regulating mt-mRNA levels. The mitochondrial degradosome is a complex composed of the RNA helicase SUV3 (also known as SUPV3L1) and polynucleotide phosphorylase (PNPase, PNPT1). It is the best-characterized RNA-degrading machinery in human mitochondria, which is primarily responsible for the decay of mitochondrial antisense RNA. The mechanism of mitochondrial sense RNA decay is less understood. This review aims to provide a general picture of mitochondrial genome expression, with a particular focus on mitochondrial RNA (mtRNA) degradation.

## Organization of the human mitochondrial genome and the basic steps of its expression

**Figure 1:**
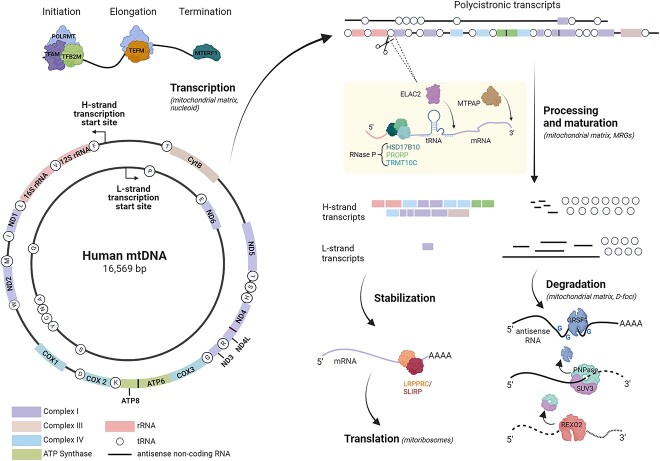
Schematic representation of human mtDNA and basic steps of mtRNA metabolism. MtDNA encodes 7 of the 43 subunits of complex I, 1 of the 11 subunits of complex III, 3 of the 13 subunits of complex IV, 2 subunits of ATP synthases, 2 ribosomal RNAs and 22 transfer RNAs. ND4L/ND4 and ATP8/ATP6 transcripts are bicistronic. Transcription of mtDNA leads to formation of two polycistronic transcripts. During the processing and maturation, individual mRNA, rRNA, and tRNA sequences are released from the primary transcript, mainly by excision of tRNAs by RNase P and ELAC2. The created transcripts undergo diverse following processes that lead to stabilization, translation or degradation. Selected proteins involved in these processes are presented. Created with BioRender.com.

Mitochondria are present in almost all eukaryotic cells, including humans. They have a genome that governs molecular mechanisms that distinguish them from all other organelles in animal cells. Mitochondrial DNA (mtDNA), unlike nuclear DNA, is distributed in foci located along the inner mitochondrial membrane, which are defined as nucleoids [1]. In human somatic cells, mtDNA is present at 1000–10 000 copies. The human mitochondrial genome consists of double-stranded circular DNA of ~16.5 kb, and the individual strands are referred to as heavy (H) and light (L) (Fig. 1). The names of the strands reflect the fact that the heavy one contains more guanine nucleotides; therefore, it can be separated from the light strand by centrifugation using a cesium chloride gradient. The expression of human mtDNA results in 13 proteins out of the 90 components of the respiratory chain and 24 RNA molecules (2 rRNAs and 22 tRNAs) that are part of the mitochondrial protein synthesis apparatus [2]. All proteins involved in the replication and transcription of mtDNA, and translation of mitochondrial mRNAs (mt-mRNAs), are encoded in the nucleus and subsequently transported into the mitochondrion [3].

Transcription of mtDNA is driven by mitochondrial RNA polymerase (POLRMT). The enzyme cannot initiate transcription independently; the other main components involved in the process are TFAM and TFB2M [4, 5]. Elongation and termination of mitochondrial transcription depend on interplay between POLRMT and TEFM or MTERF1, respectively [5, 6]. Synthesis of RNA starts in both directions within the regulatory region to produce long polycistronic RNAs that encompass almost the entire genome (Fig. 1). This primary RNA carries sequences corresponding to mRNAs, tRNAs, rRNAs, and noncoding, antisense RNAs (Fig. 1) [2]. In 1981, Ojala *et al*. [7] hypothesized the existence of a mitochondrial transcript processing mechanism, which is still widely accepted today; this mechanism is known as the “tRNA punctuation” model and is based on the observation that at least one tRNA immediately flanks almost all protein and rRNA genes (Fig. 1). It was assumed and subsequently confirmed that the first step of mtRNA processing involves the removal of tRNAs from the polycistronic transcript. This results in the formation of immature mt-mRNAs, mt-rRNAs, and mt-tRNAs, which undergo subsequent maturation steps [8]. According to the tRNA punctuation model, the transcribed tRNAs fold to form structures that act as substrates for mitochondrial RNases P and Z. RNase P induces endonucleolytic cleavage at the 5′ end of the tRNAs, whereas RNase Z (ELAC2) acts at the 3′ end [9, 10]. Mitochondrial RNase P is composed of three proteins: TRMT10C, HSD17B10 and PRORP [9]. The latter has a metallonuclease domain responsible for RNA cleavage [11, 12]. PRORP cannot carry out its enzymatic activity autonomously. Its activity strictly depends on the presence of the TMRT10C/HSD17B10 subcomplex [13]. Some mitochondrial transcripts are not flanked by tRNAs (Fig. 1). Recent studies indicate an important role for the Fas-activated serine/threonine kinase (FASTK) family members in the cleavage of noncanonical mitochondrial RNA processing sites [14].

An important feature of the human mitochondrial genome is that its transcription results in large amounts of noncoding mtRNAs, which are complementary to functional ones. This results from the unequal distribution of coding sequences between the strands of mtDNA and that both strands are almost entirely transcribed, including the noncoding regions. For most mitochondrial genes, the coding strand is the L-strand, whereas the H-strand is the template for transcription [2]. Therefore, most of the functional mtRNAs (rRNA, mRNA, tRNA) are generated by transcription of the H-strand, whereas transcription of the L-strand, in addition to the expression of eight tRNAs and one mRNA (ND6), is associated with the production of a large amount of noncoding, antisense mtRNAs [2] (Fig. 1). Under normal conditions, mitochondrial noncoding (antisense) transcripts are maintained at a very low level, which indicates that they are rapidly degraded [15–17].

Transcription of mitochondrial genes and the processing and maturation of mtRNA occur within the mitochondrial matrix. Post-transcriptional processing of mtRNA appears to be spatially organized into distinct areas known as mitochondrial RNA granules and degradosome-containing foci (MRGs and D-foci, respectively) [17–19]. One of the main components of MRGs is GRSF1, a mitochondrial protein containing three RNA-binding domains, which mainly bind guanine-rich sequences, mostly present in transcripts synthesized starting from the L-strand [18]. The decrease in the number of MRGs observed following transcriptional block suggests that the first step for MRG formation consists of the accumulation of nascent mtRNAs, which subsequently recruit the various protein components [19]. A decrease in GRSF1 levels influences mitochondria biogenesis [18]. Indeed, it has been proposed that GRSF1 plays an important role in the correct formation of mitoribosomes [18]. Moreover, the lack of GRSF1 impacts the steady-state levels of mt-mRNAs and mt-rRNAs, leads to incorrect loading of some mt-mRNAs onto mitoribosomes and affects their translation [18]. Several proteins co-localize with GRSF1 at the MRGs as revealed by thorough localization and co-immunoprecipitation studies [20]. Among them is FASTKD2, whose CRISPR- or siRNA-mediated depletion causes changes in mitochondrial transcriptome, affects mitochondrial ribosomes biogenesis and translation [20, 21]. Overall, these data indicate the role of MRGs in the mitochondrial protein synthesis and post-transcriptional processing of mt-mRNAs.

Mitochondrial RNAs undergo various modifications [22], of which the most common is the adenylation of the 3′-end. This occurs after transcription, is DNA template-independent, is catalyzed by mitochondrial poly(A) polymerase (MTPAP), and primarily involves mRNAs [23]. Ten out of eleven mt-mRNAs are adenylated, with the only exception being the mRNA encoding ND6 [24]. The role of mt-mRNA adenylation is not fully understood. For some protein-coding genes, the post-transcriptional addition of adenosine residues creates a complete termination codon, which is not present in the primary genomic sequence [2]. In this case, within the polycistronic transcript, the mt-tRNA genes are processed, leaving an incomplete stop codon, U or UA [7]. The latter is polyadenylated by MTPAP, which adds an A-tail to the 3′ end. The poly(A)-specific exoribonuclease PDE12 counteracts the activity of MTPAP by preventing spurious adenylation of mt-tRNAs and mt-rRNAs [25].

Mitochondrial noncoding, antisense RNAs are also post-transcriptionally adenylated [15]. It is not clear why poly(A) tails are added to this class of transcripts. One possibility is that the polyadenylation of mitochondrial antisense transcripts stimulates their degradation. This would be a hallmark of the bacterial origin of mitochondria, in which polyadenylation marks RNAs for degradation [26]. The fact that stable, mature mt-mRNAs, including transcripts that do not require adenylation to complete the stop codon, have poly(A) tails, indicates that polyadenylation of mt-mRNA may have other functions. For nuclear-encoded mRNAs, the length of the poly(A) tails is coupled with their steady-state levels [27]. Of note, the length of the A-tails of specific mt-mRNAs is heterogeneous and varies within the same cell type and the same transcript between different cell types [28]. Therefore, it is likely that the role of polyadenylation in the mtRNA life cycle varies depending on the length and molecular context (antisense *versus* sense transcripts). The precise role of RNA polyadenylation in mtRNA degradation and surveillance requires further study.

Interestingly, it has been described that mitochondrial transcripts can undergo the post-transcriptional addition of uridines at the 3′-end, which is known as RNA uridylation. To date, uridylation has been studied primarily in the context of nuclear-encoded RNAs [29]. Nevertheless, its role in the regulation of mtRNA decay has also been described. In *Trypanosoma brucei* mitochondria, the 3′ uridylation of mt-mRNAs stimulates their decay [30], and aberrant mitochondrial 12S rRNAs are marked for degradation by uridylation [31]. The phenomenon has also been observed in human mitochondria [15, 32], but the specific role of uridylation has not been elucidated.

## Degradation of mtRNA

Although the molecular mechanisms of human mitochondrial genome expression have been actively investigated since the 1980s, many aspects of mt-mRNA decay remain to be revealed. An intriguing and complex model of mt-mRNA decay was proposed by Liu and colleagues [33], according to which mt-mRNA degradation occurs in the mitochondrial intermembrane space (IMS) [33]. Although this hypothesis has not been verified by independent studies, such a mechanism would involve the active transport of mt-mRNA from the matrix to the IMS. However, no such pathway has been identified thus far, and the existence of RNA transport through mitochondrial membranes in mammalian cells is a controversial subject [34].

EXD2 is an exonuclease linked with mitochondrial translation [35]. The exonuclease activity of EXD2 and its ability to bind to ssRNA [35] suggested a possible role in mtRNA turnover. This has been challenged based on detailed intracellular localization studies showing its predominant association with the mitochondrial outer membrane; thus, being excluded from the mitochondrial matrix where mtRNA turnover occurs [36, 37]. Another protein with ribonuclease activity that has attracted interest in the field of mtRNA decay is LACTB2 [38], which was described as a putative mitochondrial endoribonuclease [38]. It should be noted, however, that LACTB2 activity toward ssRNA *in vitro* is very weak. Moreover, reduced expression of LACTB2 in human 293 cells caused only a modest increase in mitochondrial transcript levels, indicating that this protein does not play a major role in mtRNA turnover, if any; thus, its putative role in mtRNA metabolism requires further study.

Research performed in several last years established that the mitochondrial degradosome, a complex of SUV3 and polynucleotide phosphorylase (PNPase, also known as PNPT1), is indispensable for mtRNA degradation [16, 17, 39, 40]. SUV3 is a helicase that catalyzes the unwinding of RNA duplexes [41], whereas PNPase is a phosphorolytic exoribonuclease that catalyzes the degradation of the phosphodiester bond in RNA [42]. Chujo *et al*. proposed that the mitochondrial degradosome is responsible for the decay of mt-mRNAs [39]. However, in contrast to results obtained by Chujo *et al*., who found almost complete inhibition of mt-mRNA degradation upon degradosome silencing, we observed that the extent to which mt-mRNAs are stabilized upon PNPase or SUV3 silencing is moderate and varies significantly between individual mt-mRNAs [17]. Moreover, we found that the steady-state levels of some mt-mRNAs decreased when the degradosome components were depleted by siRNA, which is opposite to the anticipated effect of mt-mRNA decay inhibition [17]. In contrast, silencing of PNPase or SUV3 resulted in the stabilization and accumulation of mitochondrial antisense transcripts, indicating that this class of RNA is the primary substrate of the degradosome [17, 43]. The discrepancy between the data of Chujo *et al*. and our own regarding the intensity of mt-mRNA stabilization upon degradosome dysfunction likely results from the use of different methodological approaches. Because of the almost complete transcription of both strands of mtDNA, mtRNA decay should be examined in a strand-specific manner. While we measured the levels and stability of mt-mRNAs in a strand-specific manner, the others seemed not to apply this strategy. Thus, the mt-mRNA decay assays performed by Chujo *et al*. may have measured the decay of both sense and antisense transcripts without discrimination. Taken together, while the importance of the regulatory action of the mitochondrial degradosome for antisense mitochondrial transcripts is evident, the role of the complex in mt-mRNA decay requires further clarification.

In the cytosol, RNA turnover occurs within distinct granules [44]. Borowski *et al*. showed a similar mechanism in the mitochondrion using FRET and BiFC techniques [17]. The latter enabled not only the identification *in cellulo* of the mitochondrial degradosome in specific foci (named D-foci), which colocalize with mtDNA and mtRNA, but also confirmed further the link between PNPase and SUV3 [17]. Following deletion experiments, a stretch of 5 amino acids in SUV3, essential to the interaction between the two factors, was identified using *in vitro* assays [45]. Of note, it has been shown that the interaction between SUV3 and PNPase is required for the decay of mitochondrial antisense RNAs [17]. Interestingly, both PNPase and SUV3 were consistently co-immunoprecipitated with different MRG proteins [20] indicating overlap between D-foci and MRGs.

**Figure 2:**
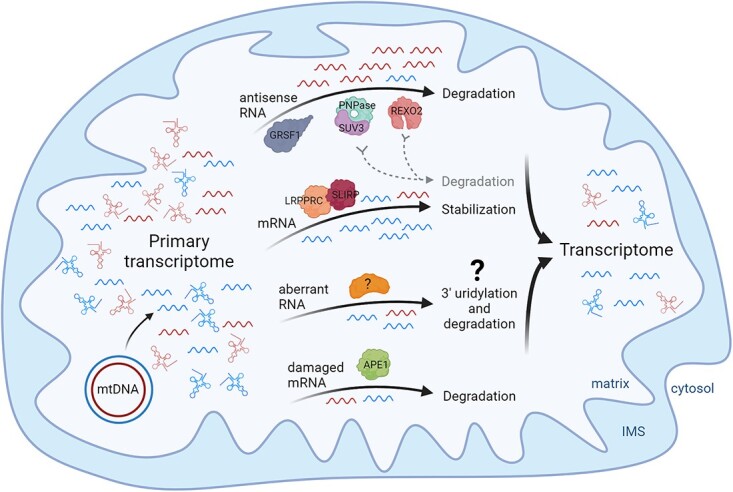
MtRNA decay and surveillance shapes the mitochondrial transcriptome. Pervasive transcription of mtDNA leads to the primary transcriptome which is then shaped by mtRNA degradation. Noncoding antisense mtRNAs are removed in degradosome-dependent manner. The complex is also involved, at least to some extent, in mt-mRNA decay. Mt-mRNAs are bound and stabilized by the LRPPRC/SLIRP complex. Some (abnormal) mitochondrial transcripts undergo uridylation. However, specific role and factors involved in this process are not known. Damaged transcripts with abasic sites are targeted by APE1 and degraded, but the exact mechanism has not been elucidated. The activity of all these decay and surveillance pathways result in formation of the mitochondrial transcriptome. Created with BioRender.com.

As a result of the degradosome activity, short oligoribonucleotides are generated [42]. It was expected that, thanks to the activity of another RNase, these short RNA species would undergo complete degradation. Indeed REXO2, an exoribonuclease with strong specificity toward short RNA substrates, was identified [46, 47]. Functional studies of REXO2 revealed that it is responsible for controlling short mtRNAs generated by mtRNA processing and the decay machinery, including the degradosome [47]. Importantly, it was also demonstrated that inhibition of REXO2 indirectly affects the activity of the degradosome by the overaccumulation of degradosome final products, which in turn, leads to the upregulation of mitochondrial dsRNA [47], a potent stimuli of the sterile inflammatory response [48].

Most biological processes are dependent upon redundant mechanisms. Disruption of a given RNA degradation pathway is at least partially compensated by the activity of other RNA-degrading mechanisms [49]. RNA degradation in yeast mitochondria proceeds primarily in a 3′ → 5′ direction; however, it was shown that a 5′ → 3′ RNA degradation pathway is also present in yeast mitochondria [50, 51]. Sequence analysis of human mtRNA degradation products observed during disrupted SUV3 function revealed that most of them were truncated at the 3′ end [15]. This indicates that degradosome-dependent mtRNA degradation proceeds from the 3′ to 5′ end of RNA, which is consistent with the results of an *in vitro* biochemical analysis of the degradosome subunits activity [42, 52]. Interestingly, 5′-truncated mitochondrial transcripts were also identified in human cells with dysfunction of SUV3 helicase [15]. Furthermore, these transcripts were detected at low levels by RNA-seq in mammalian cells with undisturbed mtRNA degradation [24]. This suggests the existence of a 5′ → 3′ RNA degradation pathway in human mitochondria, whose components and function await elucidation.

RNA degradation plays an important role in the regulation of mtDNA expression by tuning the steady-state levels of functional transcripts; however, a second important function of RNA degradation in the mitochondria is maintaining mtRNA quality (RNA surveillance). As a part of this process, not only by-products of the primary transcripts processing are removed, but also improperly processed mtRNAs and antisense mtRNAs are degraded, which ultimately shapes the mitochondrial transcriptome [15–17, 47] (Fig. 2). An emerging aspect of mtRNA decay is the control of damaged mt-mRNAs. The multifunctional protein APE1, which was previously linked to DNA repair, has been shown to target abasic sites in damaged mt-mRNAs [53] (Fig. 2). The manner in which the quality control system recognizes damaged and abnormal mitochondrial transcripts remains to be determined.

## RNA-binding proteins in mtRNA decay

The high efficiency of antisense mtRNA degradation appears to be an underestimated issue. Transcriptome analysis of various human tissues by RNA-seq revealed that 11 mt-mRNAs out of several thousand mRNAs expressed in the cell account for a significant fraction of the cellular mRNA, ranging from 5% to 30% [24]. Considering that mt-mRNAs do not have long half-lives (2–4 h for most mt-mRNAs) [39, 54], the high proportion of mt-mRNAs to total cellular mRNA indicates that they are synthesized at a high frequency. Moreover, it has been shown that L-strand transcription, which is the major source of mitochondrial antisense RNA, occurs more frequently than H-strand transcription (responsible for 10 out of 11 mt-mRNAs) [55, 56]. Thus, the level of mitochondrial antisense RNA production is expected to be quite high. Nevertheless, under normal conditions, these RNA species are maintained at a very low level, which indicates that they are removed with high efficiency. The effectiveness of this process is even more intriguing when one takes into account the fact that mitochondrial antisense transcripts are rich in guanine nucleotides.

**Figure 3:**
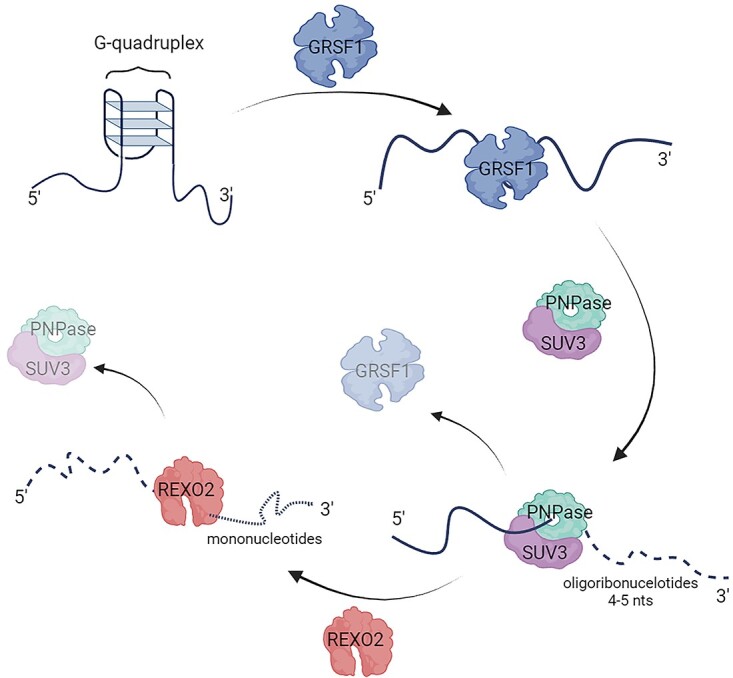
Mitochondrial antisense RNA degradosome-dependent degradation. GRSF1 binds to antisense mtRNA and melts G4 structures. Then, SUV3 helps to dissociate GRSF1 from RNA and unwinds double-stranded RNA structures, which enables degradation of the substrate by PNPase, and 4-5 nts fragments are generated. In the final stage, REXO2 removes short oligonucleotides. Created with BioRender.com.

Similar to nuclear-encoded transcripts, mitochondrial transcripts can also fold into various structures. Guanine-rich antisense mtRNAs can form G-quadruplex (G4), in which guanine residues associate by Hoogsteen hydrogen bonds. These structures are considered stable; therefore, their presence in mitochondrial antisense RNA may hamper their degradosome-dependent decay. GRSF1, a mitochondrial RNA-binding protein, was found to be associated with the active degradosome in an RNA-dependent manner [16]. Loss of GRSF1 and degradosome activity results in the accumulation of mitochondrial G4-forming RNAs. Moreover, *in vitro* RNA-binding assays and the identification of *in vivo* GRSF1 RNA substrates revealed its specificity toward G4 mtRNAs. Based on these data, a mechanism was proposed by which GRSF1 cooperates with the degradosome [16] (Fig. 3). According to the model, GRSF1 binds and melts G4s, whereas the SUV3 helicase unwinds the double-stranded structures and displaces GRSF1 from the substrate. Activity of GRSF1 and SUV3 enables the subsequent degradation of the RNA by PNPase (Fig. 3). In agreement with the proposed mechanism it was observed that GRSF1-assisted degradation of mitochondrial G4 RNAs *in vitro* is much more efficient compared with that driven by the degradosome alone [16].

LRPPRC and SLIRP are among the most well-characterized noncatalytic mtRNA-binding proteins. LRPPRC and SLIRP form a complex that is involved in maintaining mt-mRNA steady-state levels, polyadenylation, and translation [57, 58], with no obvious role in antisense mtRNA metabolism. A study using a global RNase footprinting procedure revealed that the LRPPRC-SLIRP complex modulates mtRNA folding with a strong preference for mitochondrial sense transcripts, indicating that the complex may act as a chaperone for mt-mRNAs [59]. The presence of LRPPRC is important for the existence of a nontranslated, mitoribosome-unbound pool of mt-mRNAs, indicating its role in the regulation of mt-mRNA stability [57, 58]. Of note, LRPPRC silencing has a diverse effect on the stability of individual mt-mRNAs [39], suggesting that other factors, including other mtRNA-binding proteins, such as members of the FASTK family [60], contribute to the regulation of mt-mRNA decay.

## Differential expression of the mitochondrial protein-coding genes

RNAs encoded by the same strand of mtDNA are synthesized at the same frequency; however, the levels of mature mt-mRNAs differ significantly [24, 61], which indicates that post-transcriptional processes, such as RNA decay, play an important role in the regulation of mtDNA-encoded mRNAs. To date, the underlying mechanism responsible for the variation in mt-mRNA steady-state levels has not been clarified. Of note, human mt-mRNAs form a quite divergent group. Individual mt-mRNAs differ in (*i*) the presence of 5′ or 3′ untranslated regions, (*ii*) the formation of a stop codon, (*iii*) 3′-end polyadenylation, (*iv*) the mechanism of release from the polycistronic transcript, and (*v*) translation initiation. Therefore, mt-mRNAs synthesis encoded by a given mtDNA strand is universal; however, the subsequent steps in their life cycle, including decay, may be transcript-specific.

Although an analysis of > 11 000 RNA-sequencing libraries across 36 human tissues and cell types revealed variation in the total level of mtDNA expression across tissues and between individuals, the expression profile of specific mt-mRNAs appears to be consistent [61]. For example, MT-CO2 is detected as a highly expressed gene in most transcriptomic datasets, whereas MT-ND5 or MT-ND6 are regularly observed as lowly expressed ones. Moreover, a detailed analysis of another > 120 transcriptome datasets revealed a strong positive correlation between the expression of the 13 mtDNA protein-coding genes [62]. This suggests that tissue-specific factors, which most likely act at the mtDNA transcription level, are responsible for differences in the total mtDNA expression observed across tissues and individuals. In contrast, the expression profile of steady-state levels of specific mt-mRNAs appears to be maintained by some general, nontissue-specific mechanism(s) that are active at the post-transcriptional level. Importantly, a genetic variation analysis identified a set of nuclear genes associated with mt-mRNA expression levels. This set included MTPAP and LRPPRC, suggesting that mt-mRNA polyadenylation and association of mt-mRNA with RNA-binding proteins can exert important mechanism(s) of mt-mRNA levels regulation [61].

A fascinating mechanism of mRNA regulation was proposed for human nuclear-encoded mRNAs. It has been shown that the stability of mRNAs depends on codon occurrence (not the codon bias). Wu *et al*. identified a group of “optimal” codons that promote mRNA stability [63]. This effect depends on active translation, and “the signal” is present in the coding sequence. Therefore, such a codon-dependent mechanism of RNA regulation may be particularly important for transcripts with short or no UTRs, such as mt-mRNAs. It is tempting to speculate that a similar regulatory mechanism may occur in human mitochondria.

Interestingly, a recent study by Bruni *et al*. demonstrated that mt-mRNAs unloaded to mitochondrial ribosomes are destined for degradation [64]. A reduction in mt-mRNA levels alone during depletion of the large ribosomal subunit mt-LSU was observed [64]. Under these experimental conditions, the amount of LRPPRC/SLIRP and mtDNA transcription is unchanged, suggesting that the decrease in transcripts was caused by their decay and not by a lack of synthesis [64]. In addition, a link between mitochondrial translation and mt-mRNA stability was observed when the mitochondrial release factor mtRF1 was knocked out [65] or when mitochondrial translation was inhibited by thiamphenicol treatment [54]. While the former caused a reduction in COX1 mRNA steady-state levels, but not in other tested mt-mRNAs [65], the latter increased the stability of several mt-mRNAs [54]. Whether mitoribosome/translation-dependent mt-mRNA degradation is regulated by PNPase and SUV3 or whether other factors are involved is unclear; however, the results obtained could certainly open the doors to new facets in the world of mtRNA decay, revealing the existence of mechanisms not yet described.

## References

[1] Garrido N, Griparic L, Jokitalo E. et al. Composition and dynamics of human mitochondrial nucleoids. Mol Biol Cell 2003;14:1583–96. 12686611 10.1091/mbc.E02-07-0399 PMC153124

[2] Anderson S, Bankier AT, Barrell BG. et al. Sequence and organization of the human mitochondrial genome. Nature 1981;290:457–65. 7219534 10.1038/290457a0

[3] Rackham O, Filipovska A. Organization and expression of the mammalian mitochondrial genome. Nat Rev Genet 2022;23:606–23. 35459860 10.1038/s41576-022-00480-x

[4] Yakubovskaya E, Guja KE, Eng ET. et al. Organization of the human mitochondrial transcription initiation complex. Nucleic Acids Res 2014;42:4100–12. 24413562 10.1093/nar/gkt1360 PMC3973321

[5] Miranda M, Bonekamp NA, Kühl I. Starting the engine of the powerhouse: mitochondrial transcription and beyond. Biol Chem 2022;403:779–805. 35355496 10.1515/hsz-2021-0416

[6] Minczuk M, He J, Duch AM. et al. TEFM (c17orf42) is necessary for transcription of human mtDNA. Nucleic Acids Res 2011;39:4284–99. 21278163 10.1093/nar/gkq1224 PMC3105396

[7] Ojala D, Montoya J, Attardi G. tRNA punctuation model of RNA processing in human mitochondria. Nature 1981;290:470–4. 7219536 10.1038/290470a0

[8] Rorbach J, Minczuk M. The post-transcriptional life of mammalian mitochondrial RNA. Biochem J 2012;444:357–73. 22642575 10.1042/BJ20112208

[9] Holzmann J, Frank P, Löffler E. et al. RNase P without RNA: identification and functional reconstitution of the human mitochondrial tRNA processing enzyme. Cell 2008;135:462–74. 18984158 10.1016/j.cell.2008.09.013

[10] Brzezniak LK, Bijata M, Szczesny RJ. et al. Involvement of human ELAC2 gene product in 3’ end processing of mitochondrial tRNAs. RNA Biol 2011;8:616–26. 21593607 10.4161/rna.8.4.15393

[11] Bhatta A, Dienemann C, Cramer P. et al. Structural basis of RNA processing by human mitochondrial RNase P. Nat Struct Mol Biol 2021;28:713–23. 34489609 10.1038/s41594-021-00637-y PMC8437803

[12] Reinhard L, Sridhara S, Hällberg BM. Structure of the nuclease subunit of human mitochondrial RNase P. Nucleic Acids Res 2015;43:5664–72. 25953853 10.1093/nar/gkv481 PMC4477676

[13] Reinhard L, Sridhara S, Hällberg BM. The MRPP1/MRPP2 complex is a tRNA-maturation platform in human mitochondria. Nucleic Acids Res 2017;45:12469–80. 29040705 10.1093/nar/gkx902 PMC5716156

[14] Ohkubo A, Van Haute L, Rudler DL. et al. The FASTK family proteins fine-tune mitochondrial RNA processing. PLoS Genet 2021;17:e1009873. 34748562 10.1371/journal.pgen.1009873 PMC8601606

[15] Szczesny RJ, Borowski LS, Brzezniak LK. et al. Human mitochondrial RNA turnover caught in flagranti: involvement of hSuv3p helicase in RNA surveillance. Nucleic Acids Res 2010;38:279–98. 19864255 10.1093/nar/gkp903 PMC2800237

[16] Pietras Z, Wojcik MA, Borowski LS. et al. Dedicated surveillance mechanism controls G-quadruplex forming non-coding RNAs in human mitochondria. Nat Commun 2018;9:2558. 29967381 10.1038/s41467-018-05007-9 PMC6028389

[17] Borowski LS, Dziembowski A, Hejnowicz MS. et al. Human mitochondrial RNA decay mediated by PNPase-hSuv3 complex takes place in distinct foci. Nucleic Acids Res 2013;41:1223–40. 23221631 10.1093/nar/gks1130 PMC3553951

[18] Antonicka H, Sasarman F, Nishimura T. et al. The mitochondrial RNA-binding protein GRSF1 localizes to RNA granules and is required for posttranscriptional mitochondrial gene expression. Cell Metab 2013;17:386–98. 23473033 10.1016/j.cmet.2013.02.006

[19] Jourdain AA, Koppen M, Wydro M. et al. GRSF1 regulates RNA processing in mitochondrial RNA granules. Cell Metab 2013;17:399–410. 23473034 10.1016/j.cmet.2013.02.005 PMC3593211

[20] Antonicka H, Shoubridge EA. Mitochondrial RNA granules are centers for posttranscriptional RNA processing and ribosome biogenesis. Cell Rep 2015;10:920–32. 25683715 10.1016/j.celrep.2015.01.030

[21] Popow J, Alleaume A-M, Curk T. et al. FASTKD2 is an RNA-binding protein required for mitochondrial RNA processing and translation. RNA 2015;21:1873–84. 26370583 10.1261/rna.052365.115 PMC4604428

[22] Jedynak-Slyvka M, Jabczynska A, Szczesny RJ. Human mitochondrial RNA processing and modifications: overview. Int J Mol Sci 2021;22:7999. 34360765 10.3390/ijms22157999 PMC8348895

[23] Tomecki R, Dmochowska A, Gewartowski K. et al. Identification of a novel human nuclear-encoded mitochondrial poly(A) polymerase. Nucleic Acids Res 2004;32:6001–14. 15547249 10.1093/nar/gkh923 PMC534615

[24] Mercer TR, Neph S, Dinger ME. et al. The human mitochondrial transcriptome. Cell 2011;146:645–58. 21854988 10.1016/j.cell.2011.06.051 PMC3160626

[25] Pearce SF, Rorbach J, Van Haute L. et al. Maturation of selected human mitochondrial tRNAs requires deadenylation. elife 2017;6:e27596. 28745585 10.7554/eLife.27596 PMC5544427

[26] Hajnsdorf E, Kaberdin VR. RNA polyadenylation and its consequences in prokaryotes. Philos Trans R Soc Lond Ser B Biol Sci 2018;373:20180166. 30397102 10.1098/rstb.2018.0166 PMC6232592

[27] Tudek A, Krawczyk PS, Mroczek S. et al. Global view on the metabolism of RNA poly(A) tails in yeast Saccharomyces cerevisiae. Nat Commun 2021;12:4951. 34400637 10.1038/s41467-021-25251-w PMC8367983

[28] Temperley RJ, Wydro M, Lightowlers RN. et al. Human mitochondrial mRNAs—like members of all families, similar but different. Biochim Biophys Acta 2010;1797:1081–5. 20211597 10.1016/j.bbabio.2010.02.036 PMC3003153

[29] Liudkovska V, Dziembowski A. Functions and mechanisms of RNA tailing by metazoan terminal nucleotidyltransferases. Wiley Interdiscip Rev RNA 2021;12:e1622. 33145994 10.1002/wrna.1622 PMC7988573

[30] Aphasizheva I, Aphasizhev R. RET1-catalyzed uridylylation shapes the mitochondrial transcriptome in Trypanosoma brucei. Mol Cell Biol 2010;30:1555–67. 20086102 10.1128/MCB.01281-09 PMC2832499

[31] Mattiacio JL, Read LK. Roles for TbDSS-1 in RNA surveillance and decay of maturation by-products from the 12S rRNA locus. Nucleic Acids Res 2008;36:319–29. 18032430 10.1093/nar/gkm690 PMC2248759

[32] Slomovic S, Schuster G. Stable PNPase RNAi silencing: its effect on the processing and adenylation of human mitochondrial RNA. RNA 2008;14:310–23. 18083837 10.1261/rna.697308 PMC2212247

[33] Liu P, Huang J, Zheng Q. et al. Mammalian mitochondrial RNAs are degraded in the mitochondrial intermembrane space by RNASET2. Protein Cell 2017;8:735–49. 28730546 10.1007/s13238-017-0448-9 PMC5636749

[34] Gammage PA, Moraes CT, Minczuk M. Mitochondrial genome engineering: the revolution may not be CRISPR-Ized. Trends Genet 2018;34:101–10. 29179920 10.1016/j.tig.2017.11.001 PMC5783712

[35] Silva J, Aivio S, Knobel PA. et al. EXD2 governs germ stem cell homeostasis and lifespan by promoting mitoribosome integrity and translation. Nat Cell Biol 2018;20:162–74. 29335528 10.1038/s41556-017-0016-9

[36] Hensen F, Moretton A, van Esveld S. et al. The mitochondrial outer-membrane location of the EXD2 exonuclease contradicts its direct role in nuclear DNA repair. Sci Rep 2018;8:5368. 29599527 10.1038/s41598-018-23690-y PMC5876329

[37] Park J, Lee S-Y, Jeong H. et al. The structure of human EXD2 reveals a chimeric 3′ to 5′ exonuclease domain that discriminates substrates via metal coordination. Nucleic Acids Res 2019;47:7078–93. 31127291 10.1093/nar/gkz454 PMC6648332

[38] Levy S, Allerston CK, Liveanu V. et al. Identification of LACTB2, a metallo-β-lactamase protein, as a human mitochondrial endoribonuclease. Nucleic Acids Res 2016;44:1813–32. 26826708 10.1093/nar/gkw050 PMC4770246

[39] Chujo T, Ohira T, Sakaguchi Y. et al. LRPPRC/SLIRP suppresses PNPase-mediated mRNA decay and promotes polyadenylation in human mitochondria. Nucleic Acids Res 2012;40:8033–47. 22661577 10.1093/nar/gks506 PMC3439899

[40] Toompuu M, Tuomela T, Laine P. et al. Polyadenylation and degradation of structurally abnormal mitochondrial tRNAs in human cells. Nucleic Acids Res 2018;46:5209–26. 29518244 10.1093/nar/gky159 PMC6007314

[41] Minczuk M, Piwowarski J, Papworth MA. et al. Localisation of the human hSuv3p helicase in the mitochondrial matrix and its preferential unwinding of dsDNA. Nucleic Acids Res 2002;30:5074–86. 12466530 10.1093/nar/gkf647 PMC137961

[42] Lin CL, Wang Y-T, Yang W-Z. et al. Crystal structure of human polynucleotide phosphorylase: insights into its domain function in RNA binding and degradation. Nucleic Acids Res 2012;40:4146–57. 22210891 10.1093/nar/gkr1281 PMC3351181

[43] Pietras Z, Wojcik MA, Borowski LS. et al. Controlling the mitochondrial antisense - role of the SUV3-PNPase complex and its co-factor GRSF1 in mitochondrial RNA surveillance. Mol Cell Oncol 2018;5:e1516452. 30525095 10.1080/23723556.2018.1516452 PMC6276855

[44] Anderson P, Kedersha N. RNA granules: post-transcriptional and epigenetic modulators of gene expression. Nat Rev Mol Cell Biol 2009;10:430–6. 19461665 10.1038/nrm2694

[45] Wang DD-H, Shu Z, Lieser SA. et al. Human mitochondrial SUV3 and polynucleotide phosphorylase form a 330-kDa heteropentamer to cooperatively degrade double-stranded RNA with a 3′-to-5′ directionality. J Biol Chem 2009;284:20812–21. 19509288 10.1074/jbc.M109.009605 PMC2742846

[46] Bruni F, Gramegna P, Oliveira JMA. et al. REXO2 is an oligoribonuclease active in human mitochondria. PLoS One 2013;8:e64670. 23741365 10.1371/journal.pone.0064670 PMC3669425

[47] Szewczyk M, Malik D, Borowski LS. et al. Human REXO2 controls short mitochondrial RNAs generated by mtRNA processing and decay machinery to prevent accumulation of double-stranded RNA. Nucleic Acids Res 2020;48:5572–90. 32365187 10.1093/nar/gkaa302 PMC7261184

[48] Dhir A, Dhir S, Borowski LS. et al. Mitochondrial double-stranded RNA triggers antiviral signalling in humans. Nature 2018;560:238–42. 30046113 10.1038/s41586-018-0363-0 PMC6570621

[49] Houseley J, Tollervey D. The many pathways of RNA degradation. Cell 2009;136:763–76. 19239894 10.1016/j.cell.2009.01.019

[50] Fekete Z, Ellis TP, Schonauer MS. et al. Pet127 governs a 5′ -> 3′-exonuclease important in maturation of apocytochrome b mRNA in Saccharomyces cerevisiae. J Biol Chem 2008;283:3767–72. 18086665 10.1074/jbc.M709617200

[51] Łabędzka-Dmoch K, Rażew M, Gapińska M. et al. The Pet127 protein is a mitochondrial 5′-to-3′ exoribonuclease from the PD-(D/E)XK superfamily involved in RNA maturation and intron degradation in yeasts. RNA 2022;28:711–28. 35197365 10.1261/rna.079083.121 PMC9014873

[52] Venø ST, Kulikowicz T, Pestana C. et al. The human Suv3 helicase interacts with replication protein A and flap endonuclease 1 in the nucleus. Biochem J 2011;440:293–300. 21846330 10.1042/BJ20100991 PMC4366949

[53] Barchiesi A, Bazzani V, Jabczynska A. et al. DNA repair protein APE1 degrades dysfunctional abasic mRNA in mitochondria affecting oxidative phosphorylation. J Mol Biol 2021;433:167125. 34224750 10.1016/j.jmb.2021.167125

[54] Piechota J, Tomecki R, Gewartowski K. et al. Differential stability of mitochondrial mRNA in HeLa cells. Acta Biochim Pol 2006;53:157–68. 16389406

[55] Attardi G, Chomyn A, King MP. et al. Regulation of mitochondrial gene expression in mammalian cells. Biochem Soc Trans 1990;18:509–13. 2276421 10.1042/bst0180509

[56] Blumberg A, Rice EJ, Kundaje A. et al. Initiation of mtDNA transcription is followed by pausing, and diverges across human cell types and during evolution. Genome Res 2017;27:362–73. 28049628 10.1101/gr.209924.116 PMC5340964

[57] Ruzzenente B, Metodiev MD, Wredenberg A. et al. LRPPRC is necessary for polyadenylation and coordination of translation of mitochondrial mRNAs. EMBO J 2012;31:443–56. 22045337 10.1038/emboj.2011.392 PMC3261557

[58] Sasarman F, Brunel-Guitton C, Antonicka H. et al. LRPPRC and SLIRP interact in a ribonucleoprotein complex that regulates posttranscriptional gene expression in mitochondria. Mol Biol Cell 2010;21:1315–23. 20200222 10.1091/mbc.E10-01-0047 PMC2854090

[59] Siira SJ, Spåhr H, Shearwood A-MJ. et al. LRPPRC-mediated folding of the mitochondrial transcriptome. Nat Commun 2017;8:1532. 29146908 10.1038/s41467-017-01221-z PMC5691074

[60] Jourdain AA, Popow J, de la Fuente MA. et al. The FASTK family of proteins: emerging regulators of mitochondrial RNA biology. Nucleic Acids Res 2017;45:10941–7. 29036396 10.1093/nar/gkx772 PMC5737537

[61] Ali AT, Boehme L, Carbajosa G. et al. Nuclear genetic regulation of the human mitochondrial transcriptome. elife 2019;8:e41927. 30775970 10.7554/eLife.41927 PMC6420317

[62] Wang G, Yang E, Mandhan I. et al. Population-level expression variability of mitochondrial DNA-encoded genes in humans. Eur J Hum Genet 2014;22:1093–9. 24398800 10.1038/ejhg.2013.293 PMC4135407

[63] Wu Q, Medina SG, Kushawah G. et al. Translation affects mRNA stability in a codon-dependent manner in human cells. elife 2019;8:e45396. 31012849 10.7554/eLife.45396 PMC6529216

[64] Bruni F, Proctor-Kent Y, Lightowlers RN. et al. Messenger RNA delivery to mitoribosomes—hints from a bacterial toxin. FEBS J 2021;288:437–51. 32329962 10.1111/febs.15342 PMC7891357

[65] Krüger A, Remes C, Shiriaev DI. et al. Human mitochondria require mtRF1 for translation termination at non-canonical stop codons. Nat Commun 2023;14:30. 36596788 10.1038/s41467-022-35684-6 PMC9810596

